# Smart triage: triage and management of sepsis in children using the point-of-care Pediatric Rapid Sepsis Trigger (PRST) tool

**DOI:** 10.1186/s12913-020-05344-w

**Published:** 2020-06-03

**Authors:** Alishah Mawji, Edmond Li, Clare Komugisha, Samuel Akech, Dustin Dunsmuir, Matthew O. Wiens, Niranjan Kissoon, Nathan Kenya-Mugisha, Abner Tagoola, David Kimutai, Jeffrey N. Bone, Guy Dumont, J. Mark Ansermino

**Affiliations:** 1 Department of Anesthesiology, Pharmacology & Therapeutics, University of British Columbia, 217-2176 Health Sciences Mall, Vancouver, BC V6T 1Z3 Canada; 2 School of Population and Public Health, University of British Columbia, 2206 East Mall, Vancouver, BC V6T 1Z3 Canada; 3 Walimu, P.O. Box 9924, Plot 5-7, Coral Crescent, Kololo, Kampala, Uganda; 4 Kenya Medical Research Institute/Wellcome Trust Research Programme, P.O. Box 43640-00100, Nairobi, Kenya; 5 Digital Health Innovation Lab, BC Children’s Hospital Research Institute, 948 W 28th Ave, Vancouver, BC V5Z 4H4 Canada; 6 Center for International Child Health, BC Children’s Hospital Research Institute, 948 W 28th Ave, Vancouver, BC V5Z 4H4 Canada; 7 Department of Pediatrics, University of British Columbia, Rm B2W, 4480 Oak Street, Vancouver, BC V6H 3V4 Canada; 8 Jinja Reginal Referral Hospital, Jinja, Uganda; 9 Mbagathi County Hospital, P.O. Box 20725-00202, Nairobi, Kenya; 10 Department of Obstetrics and Gynaecology, University of British Columbia, 1125 Howe Street, Vancouver, BC V6Z 2K8 Canada; 11 Electrical and Computer Engineering, The University of British Columbia, 5500 - 2332 Main Mall, Vancouver, BC V6T 1Z4 Canada

**Keywords:** Sepsis, Triage, Digital triage tool, Resource limited settings

## Abstract

**Background:**

Sepsis is the leading cause of death and disability in children. Every hour of delay in treatment is associated with an escalating risk of morbidity and mortality. The burden of sepsis is greatest in low- and middle-income countries where timely treatment may not occur due to delays in diagnosis and prioritization of critically ill children. To circumvent these challenges, we propose the development and clinical evaluation of a digital triage tool that will identify high risk children and reduce time to treatment. We will also implement and clinically validate a Radio-Frequency Identification system to automate tracking of patients. The mobile platform (mobile device and dashboard) and automated patient tracking system will create a low cost, highly scalable solution for critically ill children, including those with sepsis.

**Methods:**

This is pre-post intervention study consisting of three phases. Phase I will be a baseline period where data is collected on key predictors and outcomes before implementation of the digital triage tool. In Phase I, there will be no changes to healthcare delivery processes in place at the study hospitals. Phase II will involve model derivation, technology development, and usability testing. Phase III will be the intervention period where data is collected on key predictors and outcomes after implementation of the digital triage tool. The primary outcome, time to treatment initiation, will be compared to assess effectiveness of the digital health intervention.

**Discussion:**

Smart technology has the potential to overcome the barrier of limited clinical expertise in the identification of the child at risk. This mobile health platform, with sensors and data-driven applications, will provide real-time individualized risk prediction to rapidly triage patients and facilitate timely access to life-saving treatments for children in low- and middle-income countries, where specialists are not regularly available and deaths from sepsis are common.

**Trial registration:**

Clinical Trials.gov Identifier: NCT04304235, Registered 11 March 2020.

## Background

The global burden of pediatric mortality in low- and middle-income countries (LMICs) remains high, with 4.9 million deaths in children under 5 in 2016 [1]. Most of these deaths are due to sepsis, which is defined as the body’s response to an infection (such as pneumonia, diarrhea, or malaria) leading to organ damage and ultimately morbidity and mortality [2]. Sepsis is common worldwide, but countries in Africa report substantially higher case fatality rates (adjusted odds ratios: Africa, 7.89 [95% confidence interval (CI), 6.02–10.32]) as compared to the United States [3]. Recognizing the enormity of the global burden of sepsis (death, disability, social, and economic) led to a 2017 World Health Assembly resolution highlighting the need to prioritize prevention, recognition, and early treatment of sepsis [4].

Sepsis disproportionately affects socioeconomically disadvantaged populations in LMICs. Encouragingly, most deaths from sepsis are preventable by early detection and treatment. The majority of deaths occurring in health facilities happen occur as a result of delayed, inadequate, or inappropriate treatment. Every hour of delay in therapy is associated with an escalating risk of morbidity and mortality [5]. Simple, highly effective interventions to treat sepsis, including antimicrobials and intravenous (IV) fluids, are available at care facilities in LMICs. Yet availability and readiness to provide treatment is not always enough [6]—timely treatment may not occur because the sickest children are not prioritized.

The World Health Organization (WHO) advocates the use of Emergency Triage Assessment and Treatment (ETAT) guidelines to triage children in resource limited settings [7]. Although the ETAT system is widely adopted in LMICs, successful implementation of the guidelines into clinical practice is not always the case [8]. In LMICs, patients are frequently admitted and treated on a first-come, first-serve basis, leading to delayed care for children who are in need of urgent treatment. These priority children can receive faster treatment if every child is rapidly triaged upon arrival to identify danger and priority signs of sepsis [9]. However, sepsis is a syndrome that mimics many conditions and few health workers can confidently triage and diagnose sepsis. Evidence-based trigger tools and protocols may be useful in skilled hands, but require complex decision-making based on physiological, clinical, social, and laboratory parameters.

The purpose of this study is to develop and clinically evaluate a digital triage tool that can be used rapidly and reliably, without the need for extensive memorization or training, by frontline health workers (including nurses and non-physician clinicians) to identify critically ill children (including those with sepsis). The digital platform consists of a mobile application integrating a pulse oximetry sensor attached to the device, with embedded smart algorithms that predict a critically ill state, or level of risk in a child presenting at the hospital. The platform also includes an interactive dashboard located in strategic locations (e.g., laboratory, consultation rooms), which connects to the mobile application through a secure local network and displays the triage data to provide real-time monitoring for the physicians who manage the patients.

Over the past 10 years, we have developed, implemented, and evaluated the core technology of the Digital Triaging Platform including vital sign measurement devices (PhoneOx [10] and RRate [11]) and the mobile application and dashboard [12–14]. We have already identified candidate predictor variables using a modified Delphi process [15], and developed a risk prediction model based on the need for admission using predictors collected in over 1000 children at a Kenyan hospital [16].

## Methods/design

This protocol was developed with adherence to Standard Protocol Items: Recommendations for Interventional Trials (SPIRIT) guidelines.

### Objectives

#### Objective 1

To develop and clinically validate a digital triage tool and dashboard for improving hospital wait times to treatment administration in critically ill children, including those with sepsis.

This will involve:

- Collecting a pre-selected list of clinical variables (Additional file 1) from participants to develop a prediction model based on the need for hospital admission.
- Developing digital triage tool by implementing the derived prediction model along with ETAT triage guidelines (currently followed at study hospitals) into the Digital Triaging Platform.
- Evaluating the usability/feasibility of the digital trigger tool prior to implementation and routine use at the study hospitals.
- Determining effectiveness of the triage tool by comparing hospital wait times to treatment administration for children before and after implementation of the digital triage tool.

#### Objective 2

To validate the use of an automated Radio-Frequency Identification (RFID) method to track timeliness of interventions (see RFID system section more information).

This will involve:

- Simultaneously having trained Timekeepers track patient wait times manually, and an RFID system track patient wait times automatically.
- Comparing time data obtained from the Timekeepers with time data obtained from the RFID system to evaluate accuracy of the RFID system.
- Achieving routine use of a clinically evaluated RFID system to automate tracking of patient wait times in the study hospitals.

### Trial design

This is a pre-post intervention study concerning pediatric patients presenting to the study hospitals in seek of medical care for an acute illness. The study will take place over a period of 24 months. Participant recruitment will be initiated in April 2020 and continue for approximately 12 months. Statistical analysis, results presentation and dissemination will be conducted in the remaining months.

Study procedures can be divided into three phases: (I) pre-intervention (baseline), (II) interphase, (III) intervention (Table 1).

**Table 1:** Study Schema

**Experimental Sites:**	**Phase I Baseline (3–5 months)**	**Phase II Interphase (1–3 months)**	**Phase III Intervention (5–6 months)**	**Post-Data Collection (9–14 months)**
1. Mbagathi County Hospital, Nairobi 2. Jinja Regional Referral Hospital, Jinja	Data collection of predictors and outcomes by **study** nurses.	Model derivation, technology development, usability testing.	Data collection continues as done in Phase I by **study** nurses. Routine use of digital triage tool by **hospital** nurses.	Analysis, results presentation and dissemination.
**Control Site:**	**Phase I (12 months)**			
1. Kiambu County Hospital, Nairobi	Ongoing baseline data collection.			

#### Phase I: pre-intervention (baseline)

This will be a period of baseline data collection at Mbagathi County Hospital, Jinja Regional Referral Hospital, and Kiambu County Referral Hospital. There will be no changes to healthcare delivery procedures in the hospitals. Triage in the pediatric outpatient department will continue as per usual by hospital nurses using ETAT guidelines [7]. Research nurses will consent participants and collect health data (see Additional file 1) in the triage waiting area, where patients are waiting to be seen by the hospital triage nurses. The control site, Kiambu County Referral Hospital will participate in Phase I for an elongated period of time (see Table 1) and will not participate in Phase II or Phase III.

#### Phase II: interphase

##### Model derivation and technology development

A risk prediction model will be derived using the data collected in Phase I and implemented in a Digital Triaging Platform, along with a digitized version of the ETAT+ guidelines. The Digital Triaging Platform, including vital sign measurement devices (PhoneOx [10] and RRate [11]) and the mobile application and clinical dashboard [12–14] has already been developed and evaluated. Once the digital triage tool has been developed, it will be evaluated in potential users using simulated patient scenarios and a ‘Think Aloud’ method.

##### Usability testing and training

The digital triage tool will be evaluated for ease of interface navigation, functionality and basic workflow. A sample of 15 health workers in the study hospitals to represent the primary user groups will be selected for participation in the 60-min-long usability testing initiatives. Participants will be recruited through word of mouth as there is a very small cadre of potential participants. The objective of the training is to (1) ensure healthcare workers understand how to correctly collect and interpret patient information, and (2) to obtain feedback on the digitization of the tool. Training will use a framework that meets key international norms for testing digital tools, including, the think-aloud method and a questionnaire. Each training session will be conducted by a moderator and observer. During the evaluation, the observer will be seated next to the participant and will record user interaction with each interface, comments, errors, and duration of each task. Participants will be given 3–5 patient scenarios which will list hypothetical information to be entered into the app. This information will be designed to represent routine data collected during triage examination at the study hospitals. The moderator will provide the fictional charts to participants and instruct them throughout the tasks. During the simulated patient scenarios, participants will be asked to think aloud, in order to assess their thought process as they used the app. Participants will be specifically instructed to comment on the layout of the app screen, the dialogue on each interface, the order of tasks, and any additional observations or opinions. After learning the basics of the digital platform, the participants will be read the think aloud instructions and asked to perform the list of tasks and answer questions. The observer will complete a checklist to ensure that all tasks were completed, questions will be asked to evaluate task comprehension, and notes will be taken about whether help was needed in completing each task. At the end of the training session, participants will complete a triage tool training questionnaire to provide an understanding of the practical benefits and drawbacks of incorporating the digital triage tool into a clinical context. The questionnaire will utilize open ended questions and comment responses. From this evaluation. Responses from the survey will be anonymous. The data generated from the training phase is fictitious and will not be linked to any individual subject. Transcriptions and Think Aloud observations will be analyzed using the Framework Method [17] to assess attitudes of health workers. Responses will be transcribed and coded using NVivo [18], for the identification, examination and interpretation of emerging themes and patterns. Results from the analysis, feedback from the questionnaires, and comments on the observer checklists will be used to generate a report with suggested improvements to be shared with the quality improvement implementation team prior to Phase III.

#### Phase III: intervention

Phase III will be an interventional period involving routine use of the digital triage tool by the **hospital triage nurses** at Mbagathi County Hospital in Nairobi, Kenya, and Jinja Regional Referral Hospital in Jinja, Uganda. The digital triage tool will not replace triage policies in place at the study hospitals, but rather it will strengthen existing systems by integrating ETAT guidelines and a data-driven risk prediction model into the application. As done in Phase I, **study nurses** will consent participants and collect health data in the triage waiting area while patients are waiting to be seen by **the hospital triage nurses** (who will be using the digital triage tool). Continued collection of predictor variables will allow comparison of participant characteristics in the pre-intervention cohort and the post-intervention cohort.

## Methods: participants, interventions, and outcomes

### Study setting

This multi-site study will take place at one hospital in Uganda, and two hospitals in Kenya. In Uganda, the study will be conducted at Jinja Regional Referral Hospital in Jinja. Jinja, a city of approximately 90,000 people, is located in the Eastern region of Uganda. The Jinja Regional Referral Hospital pediatric ward admits approximately 5000 patients per year and the outpatient department sees approximately 100 patients per day. In Kenya, the study will be conducted at Mbagathi County Hospital and Kiambu County Referral Hospital, both located in Nairobi. A typical outpatient department (OPD) in the Kenyan study hospitals serves approximately 20,000 children per year and is staffed by one or two nurses who conduct triage and administer treatment, two or three clinicians who review patients and issue prescriptions, and one additional nurse who administers treatment and provides counselling to caregivers of children. The Kenyan hospitals admit approximately 2000 pediatric patients per year.

### Study sites

1. Jinja Regional Referral Hospital, Jinja, Uganda (experimental site)
2. Mbagathi County Hospital, Nairobi, Kenya (experimental site)
3. Kiambu County Referral Hospital, Nairobi, Kenya (control site)

### Eligibility criteria

**Inclusion Criteria:**

1. All pediatric outpatients seeking medical treatment of an acute illness. The lower age limit will include children aged from 0 days, and the upper age limit will be in accordance to respective hospitals’ practice for pediatric admissions (this may be 12, 15 or 19 years).
2. Informed parental/guardian consent provided.
3. Assent from children older than 8 years (Uganda site) or 13 years (Kenya sites) in addition to parental/guardian consent provided.

**Exclusion Criteria:**

1. Patients presenting to the outpatient department for elective cases (e.g. elective surgery or change of dressing) or for clinical review appointment.
2. Informed consent or assent (when applicable) not provided.

### Interventions

#### PRST (digital triage tool)

The PRST is a triage tool hosted on a digital platform that will enable frontline health workers in LMICs to identify critically ill children (i.e. severe infections, sepsis) early, so that life-saving treatment can be administered in a timely manner. The digital platform will consist of a mobile application hosted on an Android tablet integrating a pulse oximetry sensor attached to the tablet, with embedded smart algorithms that predict a critically ill state, or level of risk in a child presenting to the hospital. The platform will also include an interactive dashboard which will display the triage data to provide real-time monitoring for the clinicians who manage the patients. The dashboard will be implemented as a password protected website accessible by registered medical staff on any computer or tablet on the local network, allowing for easy and non-disruptive integration into health systems with existing electronic health records. ETAT+ criteria for triage will be incorporated as part of the digital platform (in addition to model identified from data obtained in Phase I).

#### RFID system

Timing tracking will be automated using customised RFID. RFID uses radio-frequency electromagnetic fields to identify the location of patients carrying special tags, with the help of readers located in key locations around the hospital, including the registration area, triage examination rooms, and treatment rooms. We intend to use Low Energy Bluetooth (BLE) tags that have a diameter of approximately 3 cm and weigh < 20 g. These tags will be inserted into a custom, washable arm, wrist or leg band. The tag could also be retained by the caregiver if the child was not willing to have the tag attached to them. When in close vicinity to a reader (for example, in the same room), the tag (location beacon) sends a message to a strategically located receiver to track the time at which the patient was in that precise location. The Bluetooth frequency is no different to that used by mobile phones and is not expected to interfere with other processes in the hospital. The RFID system will allow health workers to keep track of patients with ease and has the potential to increase organization in fast-paced, overburdened healthcare facilities.

### Outcomes

#### Primary outcomes

##### For model development

Hospital admission (within 5 days of assessment) status determined from hospital records, and a follow up call 7 days post discharge. This will inform development of a clinical prediction model based on need for hospital admission.

##### For effectiveness evaluation of digital triage tool

An increase of at least 20% in the proportion of critically ill children (emergency and priority cases) receiving an appropriate bundle of care within 60 min of arrival at the hospital. An appropriate bundle of care is defined as at least one of antibiotics, intravenous fluids, or oxygen as appropriate for age and clinical syndrome as determined and administered by **hospital staff**.

#### Secondary outcomes

1. Length of hospitalization determined from hospital records, and a follow up call 7 days post-discharge.
2. Final diagnosis determined from hospital records.
3. 7-day post-discharge mortality status determined from a follow up call 7 days post-discharge.
4. 7-day readmission status determined from a follow up call 7 days post-discharge.
  - Facility of readmission.
  - Treatment received during readmission.

### Participant involvement

Total study participation time is estimated to be a maximum of 60 min per participant in both the baseline (phase I) and intervention (phase III) periods.

#### Participant involvement in phase I and phase III

Potential participants will be recruited by study nurses while they are waiting in line to be seen by the hospital triage nurses. Participants can anticipate study procedures (including consent, clinical examination, and interview) to take between 35 to 50 min. The study nurses will conduct study procedures in the triage waiting area, while the participant is waiting in line to be seen by the hospital triage nurses. If it is the participant’s turn to be seen by the hospital triage nurses, study procedures will stop and there will be no interference or delays in accessing standard care. Participants will also engage in a short (10 min) follow up call 7-days post-discharge.

#### Additional procedures in phase III

In the intervention period, the hospital triage nurses will be conducting triage using the digital triage tool (which will include a digitized version of the triage guidelines in already place at the study hospitals). It is important to understand that the digital triage tool will NOT be replacing standard care, but rather it will be integrated into standard care to supplement and strengthen existing triage systems.

To reiterate, the study nurses will conduct the same study procedures (consent, clinical examination, and interview) in both the baseline (Phase I) and intervention (Phase III) periods. These procedures are conducted while the participants are waiting in line to be seen by the hospital triage nurses. The difference in Phase III is that the hospital triage nurses (that the participants are waiting in line to see) will be using the digital triage tool to triage participants.

### Sampling and recruitment

#### Sample size

##### Model development considerations

The sample size for model development is based on two components: the number of predictors expected in the final model (effective variables), *n*, and the outcome event rate, *I*. We employ the typical minimum standard of 10 events per effective variable and calculate the sample size as *N* = (*n x* 10)/*I*. Based on our previous study at a Kenyan hospital [16], we estimate the admission rate as *I =* 12%, and thus, to allow for a model with 10 predictors we require a minimum sample of 833 children.

##### Power to detect difference considerations

Based on an assumed pre-intervention rate of 27% of children who receive a bundle of care within 1 h, an assumed 20% relative increase due to the intervention, and an alpha of 0.05, an estimate of 750 children per group is needed for 80% power. The assumed pre-intervention rate is consistent with previous studies [19] and confirmed in our feasibility trial of the application and dashboard in Uganda.

##### Selected sample size

Based on the uncertainty commonly present in these smaller sample sizes [20], the desire to include the possibly of modelling non-linearities in our models (using machine learning methods) and the clinical feasibility (large case load) we plan to target a larger sample of 4000 at each site, with a minimum sample size of 1000 participants enrolled in Phase I. Based on the activity at our selected sites this would require no more than a 60% recruitment rate during a six-month period.

#### Sampling strategy

A systematic method for participant selection based on time cut-offs will be adopted to minimize sampling bias. The study nurses will be instructed to screen the first patient arriving after each time cut-off (i.e. first patient to arrive after every 30-min cut-off). If the patient is not eligible, the next patient arriving after the time cut-off will be selected, and so on. The interval between time cut-offs will reflect the time a study nurse spends with each participant (35–50 min) to maximize efficiency. When there is more than one study nurse on a given shift, the time intervals will be staggered.

## Methods: data collection, management, and analysis

### Data collection

#### Predictors, hospital outcomes, 7-day follow-up calls

All study nurses will be trained and well versed on the standard operating procedures to facilitate standardization of all measurements. Study nurses will collect data using a custom-built Android application on a Samsung Galaxy Tablet A8. The list of predictors to be collected include clinical signs and symptoms, demographic/sociodemographic data, and pregnancy/birth information (see Additional file 1). Similarly, designated study nurses will obtain hospital outcomes (see Additional file 1) from patient records and enter them into the application on the Samsung Galaxy Tablet. Study nurses will conduct 7-day follow up calls in accordance with the standard operating procedures and enter the data into the application on the Samsung Galaxy Tablet.

#### Time outcomes

Timing tracking will be automated using customised RFID (see RFID system section for details).

#### Usability testing data

Think Aloud transcriptions and observer checklists will be entered into a computer and uploaded to REDCap. The Triage Tool Training Questionnaire (Additional file 2) will be captured on paper and stored in a locked cabinet, in a locked room in our research spaces nearby the study sites.

#### Post-study healthcare worker satisfaction survey

After completion of Phase III of the study, healthcare workers that participated in the usability testing initiative will be invited to complete a Healthcare Worker Satisfaction Survey (Additional file 3). This will be used to generate a report that provides insight on the overall perception of health worker’s experiences with the digital triage tool.

### Data management

#### Data collection infrastructure

Study Nurses will collect data using a custom-built Android application, created using LambdaNative (lambdanative.org), the open-source cross-platform toolkit developed internally at BC Children’s Hospital Research institute. All data entered into the mobile application is stored in an encrypted database using the encryption cipher Rabbit. Access to the tablet and application is secured by passwords; without using the application, the encrypted files are not readable. The Masimo iSpO2® Pulse Oximeter with Micro USB Connector will be used to collect pulse oximetry and heart rate (including 30 s of raw plethysmographic data) and the Masimo Caregiver™ non-contact thermometer will be used to measure core temperature. The data collection application also contains complex error checking specific to the survey questions such as date inconsistency checks and ensures only relevant data items are collected, by dynamically hiding redundant questions.

Due to the complex nature of a large multi-center study, data will be uploaded directly from the Android tablets to REDCap (Research Electronic Data Capture, https://projectredcap.org/). REDCap is a secure web-based application designed to support data capture for research studies and it has been used for over 300,000 projects, in over 100 countries, including prior studies in Uganda [13]. Encrypted data will be stored for less than 14 days after completion of data collection on the tablets. In Kenya, the data will be directly uploaded weekly (depending on internet availability) over a secured internet connection to KTWRP servers, where it will be stored. A deidentified copy will be sent to the central study server at the BC Children’s Hospital Research institute where data will be checked for completeness and consistency with data definitions. In Uganda, the data will be sent to the central study server at the BC Children’s Hospital Research Institute. After this upload, the data on the tablets will be deleted. Each subject will be given a unique number and all data will be connected to this unique number. Using REDCap limits the amount of paper-based data, further ensuring data integrity and safety. The uploaded data will be accessible to only study team members with secure access to the server.

Data collected during follow-up interviews conducted by phone or in person will also be collected electronically and shared in the same secured manner. Personal identifiers are required for the collection of admission data and follow-up data. The data collection application contains several forms. All identifiers are collected on a single form, separate from the other forms containing non-identifying information, and stored in a separate and restricted REDCap form. Access to identifiers will be limited to those requiring this data for follow-up (i.e. only study personnel involved in follow-up or data verification). No analysts, co-investigators or principal investigators not directly involved in the follow-up or data verification will have access to this data. Access to REDCap will require 2-way authentication: in addition to the normal password process, a secure code (sent via SMS to the user) will be required for access to this data.

Paper based data collection items include consent forms and research assistant field notes (which do not contain identifiers). These will be stored in a locked cabinet, in a locked at our research spaces next to each study site.

#### Health intervention infrastructure

The bundle of care during the intervention will include triage in the OPD using an additional custom Android web-app, running locally on the device with no required Internet connection. As with the data collection app, this application will be password protected and data will be stored encrypted on the device. Following each triage, the triage data will be sent to a local low-cost Unix server Soekris box based in a secure room at each site. Data will be sent through encrypted HTTPS requests to server-side PHP scripts, which insert it within MySQL tables.

The clinician dashboard that will be used to clinically manage all children in the OPD who have been triaged will be implemented as a password protected website accessible by registered medical staff on any computer or tablet on the local network. As with the triage app, this website runs completely locally, independent of an outside Internet connection and is not accessible from off-site. The dashboard is implemented using the Laravel (https://laravel.com), a PHP web framework, which queries the MySQL server tables securely. Personal identifiers will be collected in the triage app and sent to the server for display on the dashboard as is necessary for correct identification of patients, but such information will never leave the hospital site. As the study hospitals already have computers in their OPDs, use of the clinical dashboard will not be extra work for the hospital staff or interfere with other tasks.

### Statistical analysis

#### Model development

The predictive model for severe infection and sepsis in children will be developed based on the need for hospital admission of 24 h or longer. To find the best performing, and most parsimonious model, a variety of model building techniques will be compared. These will include but are not limited to: stepwise regression based on Akaike’s Information Criterion (AIC), penalized regression via elastic net, and random forest [21]. Models will be compared based on the area under the receiver operating curve, and the specificity achieved at high (80–90%) sensitivity thresholds. Models within 10% of the best performing model will be considered. Final model selection will depend on parsimony, availability of predictors across sites (based on resources, cost and feasibility of collection), discrimination and calibration. All model comparisons will take place within an internal validation resampling framework such as cross validation or bootstrapping. Risk thresholds to stratify participants into triage categories (emergency, priority, and queue) be selected based on sensitivity analysis and expert opinion. To account for geographical differences and disparities in disease prevalence (high malaria prevalence in Jinja, Uganda), we will use net reclassification improvement to optimize and re-calibrate (if required) the prediction model [22].

#### RFID system validation

We will validate the RFID technology against a human timekeeper control for the time to bundle of care outcome by investigating bias and accuracy using a Bland Altman analysis. The RFID technology will be considered as interchangeable with a human timekeeper if the 95% limits of agreement fall within +/− 5 min. We chose this cut off as delays in bundle of care delivery below this threshold is unlikely to be of clinical significance. We will use the first 100 pairs of RFID and human measurements to calculate a mean difference and standard deviation of difference between the two measurements. Using these two values and a clinically acceptable limit of 5 min, with an alpha of 0.05 and beta of 0.2, we will calculate the sample size required for the Bland Altmann analysis by methods previously described [23]. If the 95% limits of agreement fall outside of 5 min, the same Bland Altman analysis will be done for the patient arrival time and time of bundle of care delivery; these comprise of the start and end time components of the time to bundle of care outcome. These secondary analyses serve to ascertain which of the two components contains the larger discrepancy between RFID and human measurement. Should these secondary analyses be necessary, implementation issues in the RFID technology will be brainstormed in focus groups with the technology team and addressed on the ground, and we will repeat the same validation process as described. After successful logistical improvements and validation of the RFID technology to within 5 min of a human timekeeper, we will utilize the RFID technology for data collection with a human timekeeper as backup as needed.

#### Outcomes analysis

We will be conducting our primary analysis using a difference in differences method. For our primary analysis, a logistic regression model for receipt of bundle of care within 1 h will be fit including: 1) phase of study (pre vs. post PRST) and 2) group (intervention versus control) as independent dummy variables and 3) their interaction. The interaction term corresponds to the difference in log-odds between groups in baseline vs intervention and it’s exponent as the ratio of odds ratios. We will summarise this difference by estimating the marginal effect (risk difference) of phase in both the control and intervention groups. The difference of these marginal effects is the so-called ‘difference in difference’ for a binary outcome [24]. Confidence intervals will be based on the delta-method, or bootstrapping, where appropriate. A similar approach will be used for binary secondary outcomes (mortality, readmission), or quantile regression for median time to bundle of care, a non-parametric outcome.

Subgroup analyses specified a priori include Ugandan vs. Kenyan intervention site, young infant (under 2 months old) vs. children under 5 vs. older children, and presentation during day-shift hours versus night-shift hours (if applicable). We will also perform subgroup analyses on each triage category (emergency, priority, queue); patients from the pre-intervention phase will be retroactively categorized into these subgroups for this analysis. These subgroup analyses will be conducted with the inclusion of a three-way interaction between said variable, phase, and group. Statistical significance will be based on a likelihood ratio test comparing models with and without this term. This three-way interaction term would represent potential interactions between different subgroups and our difference in odds ratio obtained in our primary analysis, and results will again be presented on the risk difference scale based on marginal effects. This method of subgroup analysis sometimes termed a difference in difference in differences method.

Although none are expected, if there are any time varying confounders identified during the study that differ between intervention and control (health system or policy changes, natural events, changes in patient populations etc.) they will be included in above models as sensitivity analyses.

As we are continually collecting data regarding predictors for admission alongside the clinical team, there is the possibility that our research activities may interact with the efficiency of delivering the sepsis bundle of care. Although the possibility of such interaction is minimized by our use of dedicated research personnel who would not approach patients who are actively receiving clinical care, any such interactions could potentially limit the external validity of our study to non-research-controlled settings. Therefore, to assess this potential interaction, we will pause the collection of predictor data 1 month before and after implementation of the PRST program. This pause will also occur in the control group in order to replicate any bias caused by changes in our primary outcome attributable to the pause rather than the PRST program. To investigate the potential for this bias, we will treat the collection or non-collection of predictors as a dummy variable for subgroup analysis using the difference in difference in differences method described above.

All analyses will be conducted using R statistical software [25], and an alpha level of 0.05 will be considered statistically significant for all outcome analyses.

## Methods: monitoring

### Data monitoring

Internal monitoring of study processes will be done regularly by the study coordinators at each hospital site. During monitoring, data and consent forms for 10% of enrolled participants will be reviewed for compliance. Retraining of study staff will be done to correct for any inconsistencies noted during monitoring and follow-up will be subsequently done by the study coordinator to ensure compliance. Given that the digital triage tool is community and health system level intervention, a data monitoring committee was not deemed necessary.

Data completeness will be continuously monitored using daily, weekly and monthly reports. Accuracy of data will be verified using an audit of 5% of cases by a data manager who will not be involved in enrolling subjects. Preliminary data quality checks and analysis will be performed throughout the data collection stage to ensure that data collected is valid and secure. These data quality checks will include checking the quality of pulse oximetry waveform data via implementing a Signal Quality Index (SQI) algorithm and checking for completeness and validity of input data. Further data quality checks will inspect the number of patients enrolled, the number of patients admitted, and the timed outcome data. Upon completion of data collection, a summary of the data collected will be compiled and will be discussed by the investigators and the study nurses to ensure that data is clean, correct and useful.

## Ethics and dissemination

### Research ethics approval/protocol amendments

Ethical approval has been obtained from Makerere University School of Public Health (MUSPH) Higher Degrees, Research and Ethics Committee, Kenya Medical Research Institute (KEMRI) Scientific & Ethics Review Unit (SERU), and The University of British Columbia/ Children’s and Women’s Health Centre of British Columbia Research Ethics Board (UBC C&W REB). MUSPH provided approval for the study to be conducted at Jinja Reginal Referral Hospital in Jinja, Uganda. KEMRI SERU provided approval for the study to be conducted on behalf of both Mbagathi County Hospital and Kiambu County Referral Hospital in Nairobi, Kenya. A copy of the protocol proposed informed consent forms, other written participation information, and any proposed advertising material will be submitted for written approval. The investigators will submit and, where necessary, obtain approval from the Institutional Review Board (IRB) for any major protocol amendments and changes to the informed consent document. The study team are responsible for assuring that this protocol and the associated informed consent documents and study-related documents are approved prior to implementation of the protocol. Any major amendments to the protocol, informed consents, or other study- related documents must be approved by the IRB prior to implementation.

### Informed consent and assent

**All consent materials will be approved by all three IRBs listed in the section above prior to use.**

The study nurses will be responsible for screening and consenting participants. This would be the norm in Kenya and Uganda. The study nurses will be certified and trained to ensure that the caregiver has a complete understanding of the consent processes, the consent form, and that the caregiver is of legal age (18 years old) and competent to provide consent.

In obtaining and documenting informed consent, the site investigators and their designees will comply with applicable local and domestic regulatory requirements. This clinical study will have a paper-based informed consent form (ICF) for enrollment developed for local use that are in accordance with applicable guidelines. The consent form will include the purpose of the study, a description of the procedures to be followed and the risks and benefits of participation. The informed consent process will give individuals all of the relevant information they need to decide whether to participate, or to continue participation, in this study. Potential research participants’ caregivers will be encouraged to ask questions and to exchange information freely with the study team. Participants will be informed on who to contact (Principal Investigator) should they have any questions during, or after the study period. If the caregiver providing consent is illiterate, an independent witness will be present to verify to the caregiver that all the information read aloud is contained in the ICF. In this instance, both the caregiver and witness will sign the ICF. The caregiver will voluntarily sign, thumbprint and date (thumbprint acceptable if illiterate who will also require a witness) the consent form if they wish to participate in the study and will be provided with a copy of the consent form. A signed and dated copy of the consent form will be kept in the documentation file at all times.

There will be consent forms specific to Phase I and Phase III. Assent will be sought for children aged 13 and older in Kenya, and for children aged 8 and older in Uganda. The lower age limit for assent was selected based on site-specific requirements, which differ in Kenya and Uganda. Consent will also be sought for participation in usability testing initiatives, the Triage Tool Training Questionnaire, and the Healthcare Worker Satisfaction Survey. Consent forms will be made available in English and the local language of the catchment population at each study site, which is Kiswahili in Kenya and Luganda in Uganda.

In emergency cases, consent will be deferred until the child is stable and study procedures will only begin after initiation of emergency treatment. Study procedures will not delay or interfere with access to standard care. If consent is not granted, the data will be deleted. This deferred consent procedure is to avoid introduction of bias by neglecting to obtain data from the most severely ill children while avoiding delays in providing care to the child. This procedure has been used in previous studies involving children with severe illness, including use by the Fluid Expansion As Supportive Therapy (FEAST) study [26].

### Confidentiality

Data will be stored and distributed using password protected locations and secure data transfer. All data entered into the digital triage tool is stored in an encrypted database using the encryption cipher Rabbit. Access to the digital tool is secured by passwords and without using the application, the encrypted files are not readable. Encrypted data will be stored for a maximum of 2 weeks on the tablets before being directly uploaded and stored stored in a secure research server at MUSPH (Uganda), or KEMRI (Kenya), and UBC. After this upload, the data on the devices will be deleted. Data will be entered into the digital triage tool using a REDCap electronic data collection form. REDCap is a secure, web-based application designed to support data capture for research studies. Each subject will be given a unique number and data will be connected to this unique number. Using REDCap limits the amount of paper-based data; further ensuring data integrity and safety. The uploaded data will be accessible to any team member with secure access to the server, including investigators from MUSPH, KEMRI, and UBC. Standard operating procedures will be implemented for the security of data, physical devices and networks. All research staff will be well trained and understand that privacy and confidentiality are imperative. All paper forms used for consent will not contain the study number. These forms will be stored in our research spaces near the study sites under lock and key, for the duration of the study and for any additional time required by the local and/or national guidelines at the time.

### Risks/adverse events

We do not anticipate any adverse events directly attributable to the study. The most significant risk in this study is a small delay in treatment administration due to inappropriate triage by the digital triage tool. This delay would not be significantly different from the baseline standard of care. The risk of an adverse event is minimal as the digital triage tool will only be used to guide the frontline health workers in identifying critically ill children in need of prompt assessment. All subjects will still be assessed by a healthcare provider regardless of triage status assigned by the digital tool.

All children enrolled into this study will receive standard care according to local, regional and national guidelines. No study procedures will take place which in any way interfere with the prescribed care. Study procedures will be delayed, when necessary, to ensure that these procedures will not impact recommended care.

Other potential risks include:

#### RFID tagging

The RFID tag is a new and unfamiliar piece of technology that needs to be worn or attached to participants who may not feel comfortable with it. We will ensure that participants/caregivers are fully aware of the purpose of wearing the RFID tag and how the system works. Further, the tag is a tiny piece of plastic that can be easily concealed and will not cause any discomfort to participants. This has been discussed with our local PI’s and local ethics committees in Kenya and Uganda. This has been approved by both the local and national ethics committees.

#### Blood sampling

Blood sampling will only be conducted when clinically indicated are already routine hospital procedures. This will not be done by research staff for research purposes, but rather we will be ensuring the resources are available to the hospital nurses to do this testing if indicated. These will be communicated by the consultant during the consultation that follows the triage process.

#### Coercion

Caregivers may feel coerced to enroll in the study in order to receive care for their child within a research setting, which may be perceived as of a higher quality than the standard of care. This will be minimized by ensuring that study nurses emphasize that the child will receive medical care whether enrolled in the study or not.

### Access to data

After the study period, a de-identified copy of the data will be prepared for deposition in a repository with open access with proper governance mechanisms. We will make every effort to prevent re-identification of subjects by coding data that has the potential of being identifiable. For example, we will convert all dates into meaningful decimal numbers (date of birth into days since birth and date of recruitment will be reduced to month of recruitment) and all locations will coded into data that is useful but not specific (such as address converted to distance and direction from facility). We will ensure that data elements with small numbers of subjects (less than 10) will be coded or lumped to avoid identification. The de-identified study data will be made publicly available using the Harvard Dataverse (https://dataverse.harvard.edu/), which is the data repository for KWTRP, and a URL will be made accessible. To enhance visibility, sharing and collating datasets with other collaborating sites for increased usability/re-use, de-identified will also be shared availed to reputable data hosting service such as the INDEPTH Data Repository (http://www.indepth-ishare.org/index.php/home), or through the newly established Pediatric Sepsis CoLab (sponsored by the World Federation of Pediatric Critical and Intensive care Societies). Sharing and access will be managed and subject to institutional agreements (KEMRI and UBC) that will set terms for how requests and access will be managed. We will ensure that a rigorous data governance structure is used by the data hosting service. The distribution will only occur with agreement from Principal Investigators and the investigators at all of the study sites. Data will also be shared through peer reviewed publications and through the Wellcome Trust open data initiatives. Data will be made available within 12 months following completion of the study.

### Presentation and dissemination of results

#### Results presentation

The results of this research will be primarily presented through at least one published manuscript with detailed description of the background, methods, results, and conclusion. The specific format and details of this manuscript will be in accordance with the requirements of the publishing journal. All usage of data for publications and other forms of data dissemination will occur jointly between collaborative institutions and include authors from both sites in all publications.

#### Dissemination

Results will be disseminated to local hospital teams and key stakeholders such as at the annual conference held by the Kenya Pediatric Association (KPA). A robust knowledge translation approach is a key aspect of our transition and scale-up. An integrated equity-oriented cascade approach [27] will be used to guide knowledge translation across the duration of the project. We will engage the “6 Ps” stakeholder groups (public, patients/ caregivers, policymakers, practitioners, press, and private sector), all of which are critical to the successful outcome of our project. Our key knowledge translation activities will employ a rich range of communication channels and will be multifaceted: academic, governmental, policy-driven, and public-facing. Methods of dissemination will include social media, radio, websites, progress reports, workshops, community meetings, executive summaries, technical reports, verbal presentations to key stakeholders, peer-reviewed scientific publications and conference presentations. All relevant reports, publications and data will be freely available online.

## Discussion

Our team has demonstrated that simple and affordable technology can circumvent the lack of training and assist in identification and follow-up of high-risk children following treatment for infections [28]. Our technology aims to improve the quality of care, defined as the degree to which health services for individuals and populations increase the likelihood of desired health outcomes and are consistent with current professional knowledge. Quality of care is suboptimal in many LMICs, where more than 8 million people die yearly from conditions that should be treatable by the health system [9]. Nearly 60% of these deaths are from conditions that should respond to appropriate health care but occur due to poor-quality care [29].

The PRST will facilitate data-driven and evidence-based improvements to the quality of care provided to children with critical illness (i.e., sepsis). The digital platform consists of a mobile application integrating a pulse oximetry sensor attached to the device, with embedded smart algorithms (which include ETAT guidelines) that predict a critically ill state, or level of risk for children presenting to the outpatient department. The platform also includes an interactive dashboard located in strategic locations (e.g., laboratory, consultation rooms), which connects to the mobile application through a secure local network and displays the triage data to provide real-time monitoring for the physicians who manage the patients. Additionally, an RFID method for tracking patients will be used to automatically collect data on the timeliness of interventions. The dashboard and RFID tracking system will increase data accuracy and completion, enhance communication and empower health workers, and improve resource allocation.

Using the PRST, each child presenting to the healthcare facility is rapidly triaged based on clinical symptoms, signs, and vital signs, which are captured by front-line health worker in less than 5 min. Based on these data, smart algorithms in the mobile application assign a level of risk (emergency, priority, queue) to each child. Following identification of risk, we target the expedited administration of evidence-based, low-cost interventions such as antibiotic, fluid, and oxygen therapy. The timely administration of this life-saving bundle of care is driven by the triage data displayed on the dashboard.

The main objective of the PRST is to enable frontline health workers to recognize the most urgent children more rapidly and allocate necessary resources more efficiently. We designed the PRST specifically for use in low-resource settings. The mobile application, integrated pulse oximetry sensors, and dashboard are easily accessible, affordable, robust to internet and power interruptions (system includes a secure local network that does not require an active internet connection) and do not rely on sophisticated expertise to operate.

Performance of the PRST may vary depending on geographical location, season, and with different disease prevalence and severity. Standardized measurements will be collected at sites in both Kenya and Uganda in order to explore these differences and optimize performance of the PRST at each site. External validation will be necessary to determine generalizability in other LMICs.

## Supplementary information

**Additional file 1.** Smart Triage Data Dictionary.

**Additional file 2.** Triage Tool Training Questionnaire.

**Additional file 3.** Healthcare Worker Satisfaction Survey.

## Data Availability

Within 12 months of study completion, de-identified study data will be made publicly available. The de-identified study data will be made publicly available using the Harvard Dataverse (https://dataverse.harvard.edu/), which is the data repository for KWTRP, and a URL will be made accessible. To enhance visibility, sharing and collating datasets with other collaborating sites for increased usability/re-use, de-identified will also be shared availed to reputable data hosting service such as the INDEPTH Data Repository (http://www.indepth-ishare.org/index.php/home), or through the newly established Pediatric Sepsis CoLab (sponsored by the World Federation of Pediatric Critical and Intensive care Societies).

## Abbreviations

AIC: Akaike’s Information Criterion
BC: British Columbia
BLE: Low-energy Bluetooth
CI: Confidence interval
ETAT: Emergency Triage and Treatment
FEAST: Fluid Expansion As Supportive Therapy
HTTPS: Hypertext Transfer Protocol Secure
ICF: Informed consent form
IEC: Independent Ethics Committee
INDEPTH: The International Network for the Demographic Evaluation of Populations and Their Health
IRB: Institutional Review Board
IV: Intravenous
KEMRI: Kenya Medical Research Institute
KPA: Kenya Pediatric Association
KWTRP: KEMRI-Wellcome Trust Research Programme
LMIC: Low-and middle-income countries
MUSPH: Makerere University School of Public Health
OPD: Outpatient Department
PHP: Hypertext Preprocessor
PRST: Pediatric Rapid Sepsis Trigger
REDCap: Research Electronic Data Capture
RFID: Radio-Frequency Identification
SERU: Scientific & Ethics Review Unit
SMS: Short Message Service
SQI: Signal Quality Index
UBC: University of British Columbia
UBC C&W REB: University of British Columbia/ Children’s and Women’s Health Centre of British Columbia Research Ethics Board
URL: Uniform Resource Locator
USB: Universal Serial Bus
WHO: World Health Organization
