# Supplementary material for: Smart triage: triage and management of sepsis in children using the point-of-care Pediatric Rapid Sepsis Trigger (PRST) tool
Source: BMC Health Serv Res. 2020 Jun 3;20:493. doi: 10.1186/s12913-020-05344-w (PMC7268489; doi:10.1186/s12913-020-05344-w)
Supplement: Supplementary file 1 — Additional file 1. Smart Triage Data Dictionary. [file 12913_2020_5344_MOESM1_ESM.pdf]

SMART
TRIAGE

Smart Triage: Triage and management of sepsis in children using the point-of care Paediatric Rapid Sepsis Trigger (PRST) tool

Data Dictionary

To be implemented a digital platform used for data collection

Colour Key:

**Form Title**
**Heading Title**
**First order condition in branching logic**
Variables triggered by the condition
**Second order condition in branching logic**
Variables triggered by the condition
**Third order condition in branching logic**
Variables triggered by the condition

Field Name in REDCap	Variable Name	Standardized Prompt	Choices
**Initial Contact**			
**Patient Information**			
study_id	Study ID	Study ID	
eligible	Eligible	Is the child eligible to participate in this study?	0, No<br>1, Yes
**IF eligible = no**			
not_eligible	Reason for ineligibility	Why is the participant NOT eligible?	1, Non-infectious Cause<br>2, Special Clinic<br>3, Immunizations<br>4, Scheduled Appointment
**IF eligible = yes**			
enrolled	Enrolled	Is the child enrolled in the study?	0, No<br>1, Yes
**IF enrolled = no**			
not_enrolled	Reason for no Enrollment	Why was the child not enrolled in the study?	1, Declined consent<br>2, Insufficient time<br>3, Language barrier<br>4, No explanation<br>99, Other
**IF not_enrolled = other**			
not_enrolled_other	Other reason for no enrollment	If reason for no enrollment is 'other', please specify.	
sd	SmartDischarges Study	Did the child participate in the SmartDischarges study?	0, No<br>1, Yes
**IF sd= yes**			
sd_id	SmartDischarges Study ID	What is the child's SmartDischarges study ID number?	
arrival_time	Hospital Arrival Time	Enter the child's hospital arrival time. This can be found on a sticker, paper slip, or notebook which has been given to the caregiver.	
hosp_id	Hospital ID	Hospital ID	
first_name	First Name	First Name	
last_name	Last Name	Last Name	
date_enrolled	Date of Presentation	Current Date	
exam_time	Time of Initiation of Examination/interview (Current Time)	Current Time	
sex	sex	Enter biological sex.	1, Female<br>2, Male
dob_known	Precise date of birth known?	Ask: Do you know exactly what day the child was born?	0, No<br>1, Yes
**IF dob_known = yes**			
dob	Date of birth	Enter exact date of birth	
**IF dob_known = no**			
yob	Estimated year of birth	Enter estimated year of birth	
mob	Estimated month of birth	Enter estimated month of birth	
agecalc	Calculated age at admission.	Age (months)	

**Vital Signs**			
spo2	Oxygen saturation	Measure peripheral oxygen saturation using the mobile pulse oximeter (See SOP).	
sqi	Signal Quality Index	Enter the signal quality index achieved (See SOP).	
hr	Heart Rate	Record the patient's heart rate from previous pulse oximetry measurement (See SOP).	
rr	Respiratory Rate	Measure respiratory rate using the app provided (See SOP).	
temp	Temperature	Measure and record axillary temperature (See SOP).	
vitals_complete	Time at completion of vitals (calculated)	Time at completion of vital sign measurements.	
oxygen	Supplemental oxygen	Is the child receiving supplemental oxygen?	0, No<br>1, Yes
**IF oxygen = yes**			
o2_modality	Modality of oxygen delivery	What supplemental oxygen supply is the child receiving?	0, Room air<br>1, Nasal cannula 2, Face mask<br>3, Non rebreather 4, Ventilated<br>5, Other
**IF o2_modality = other**			
o2_other	Other modality of oxygen delivery	What supplemental oxygen is the child receiving?	
**IF o2_modality = nasal cannula, face mask, non-rebreather mask**			
o2_flow	Flow of oxygen delivered (L/min)	What the oxygen flow in L/min?	
**Anthropometrics**			
weight	Weight (measured in kg)	Measure and record the child's weight in kg (see SOP).	
height	Height (measured in cm)	Measure and record the child's height, or length if child is under 2 years old in cm (see SOP).	
muac	Middle upper arm circumference (measured in mm)	Measure and record the child's mid-upper arm circumference in mm (See SOP).	
**Danger Signs**			
complaint	Primary complaint (reported)	Ask: What is the main reason for bringing the child here today? Read out options.	1, Cough<br>2, Difficulty breathing<br>3, Nasal congestion<br>4, Skin Rash<br>5, Abscess<br>6, Fever<br>7, Eye pain/redness<br>8, Ear pain/discharge<br>9, Diarrhea<br>10, Constipation<br>11, Vomiting<br>12, Nausea<br>13, Abdominal Pain<br>14, Pain on urination<br>15, Jaundice<br>16, Lethargy (less energy than normal, unable to eat or drink)<br>17, Malaise (generalized aching)<br>18, Convulsions

			19, Headache<br>20, Swelling (of any body part)<br>21, Anorexia<br>22, Trauma<br>23, Other
**IF complaint = other**			
complaint_other	Other primary complaint (reported)	Ask: What is the main reason for bringing the child here today? Enter **ONE** complaint only.	
**Airway and Breathing**			
cyanosis	Central cyanosis (observed)	Are the child's lips or tongue a blue or dark blue color?	0, No<br>1, Yes
difficulty_breath_rep	Difficulty breathing (reported)	Ask: Compared to when the child was well, is the child having difficulty breathing?	0, No<br>1, Yes
difficulty_breath_obs	Difficulty breathing (observed)	Is the child having difficulty breathing compared to a well child?	0, No<br>1, Yes
**Circulation**			
cap_refill	Capillary refill time > 3 seconds (upper limb)	Apply pressure to a thumb or finger for 3 seconds to blanch it. Does it take more than 3 seconds to return to original pink color after you let go? (See SOP)	0, No<br>1, Yes
**IF cap_refill = yes**			
cool_skin	Cool peripheries (observed)	Are the child's hands cold compared to the chest or trunk?	0, No<br>1, Yes
pulse_r	Weak or absent radial pulse (observed)	Does the child's radial pulse feel weak or absent?	0, No<br>1, Yes
**IF pulse_r = yes**			
pulse_c	Weak or absent central pulse (observed)	Does the child's central pulse feel weak or absent?	0, No<br>1, Yes
**Coma/Convulsions**			
alert	Alert (observed)	Is the child alert? (see SOP).	0, No<br>1, Yes
**IF alert = no**			
avpu	AVPU (observed)	Assess and select consciousness level using the AVPU scale (See SOP).	0, Alert<br>1, Responds to voice<br>2, Responds to pain<br>3, Unresponsive
convulsions	Convulsions (reported, history of)	Ask: Has the child had convulsions before? (See SOP).	0, No<br>1, Yes
convulsions_now	Convulsions during assessment, (observed)	Was the child actively convulsing during this assessment?	0, No<br>1, Yes
**Dehydration/GI/GU**			
vomiting	Vomiting (reported)	Ask: Does the child throw up everything they eat or drink?	0, No<br>1, Yes
diarrhoea	Diarrhoea (reported)	Ask: Has the child had diarrhoea 3 or times per day since getting sick?	0, No<br>1, Yes
**IF diarrhoea = yes**			
chronic_diarrhoea	Persistent Diarrhoea (reported)	Ask: Did the child have diarrhoea for more than two weeks?	0, No<br>1, Yes
dysentery	Dysentery (reported)	Ask: Has the child had blood in their stools?	0, No<br>1, Yes
**IF HR > 120 for age < 5, or HR > 100 for age ≥ 5 or diarrhoea = yes**			

skin_pinch	Skin pinch (observed)	Pinch the child's skin on the lower abdomen until it is tented, then release it. Does it take longer than 2 seconds to return to baseline?	0, No<br>1, Yes
sunken_eyes	Sunken eyes (observed)	Does the child's eyes appear to sink into their sockets?	0, No<br>1, Yes
**IF sunken_eyes = yes**			
sunken_eyes_rep	Sunken Eyes (reported)	Ask: Does the child's eyes appear to sink into their sockets compared to when the child is well?	0, No<br>1, Yes
no_tears	No tears when crying (reported)	Ask: Has the child stopped making tears when crying?	0, No<br>1, Yes
dry_mouth	Dry oral mucosa (observed)	Does the child's oral mucosa (not lips) appear dry?	0, No<br>1, Yes
**IF age < 18 months**			
dep_fontanelles	Depressed fontanelle (observed)	Does the child's fontanelles appear depressed or sunken?	0, No<br>1, Yes
**Priority Signs**			
**Trauma**			
trauma	Major trauma (observed)	Has the child suffered trauma needing a designated trauma team or urgent surgery?<br>OR Does he/she have a penetrating injury, pelvic or long bone fracture, or head or neck injury?	0, No<br>1, Yes
burn	Burns (observed)	Has the child suffered a burn resulting in skin breakdown?	0, No<br>1, Yes
poison	Poisoning (reported)	Ask: Has the child been poisoned from swallowing a chemical or drug?	
**IF poison = yes**			
poison_substance	Potentially Poisonous Substance (reported)	Ask: What did the child swallow?	1, Drug (medicine)<br>2, Chemcial (organophosphate)<br>3, Other
**IF poison_substance = other**			
poison_other	Potentially Poisonous Substance (other)	If poisonous substance is 'other', please specify.	
**IF poison_substance = drug or chemical**			
poison_name	Name of Potentially Poisonous Substance	If known, enter the name of the drug or chemical that was consumed.	
sev_pain	Severe Pain (observed)	Does the child seem to be in severe pain?	0, No<br>1, Yes
**Circulation**			
pallor	Palmar Pallor (observed)	Is the child pale at their palms compared to their caretaker?	0, No<br>1, Yes
**Neurological**			
irritable	Irritability/restlessness (observed)	Has the child been crying uncontrollably throughout the interview, even before you approached them?	0, No<br>1, Yes
**IF age < 12 months**			
cant_drink	Inability to drink/breastfeed/feed for longer than 6 hours (reported)	Ask: Has the child been too sleepy or tired to breastfeed, drink, or eat for more than 6 hours?	0, No<br>1, Yes<br>98, Don't know

**IF age ≥ 12 months**			
cant_sit	Inability to sit or stand for longer than 6 hours (reported)	Ask: Has the child been too sick to sit or stand for longer than 6 hours?	0, No<br>1, Yes<br>98, Don't know
**Respiratory**			
**IF: difficulty_breathing_rep = yes OR difficulty_breathing_obs = yes OR RR > 40 OR spo2 < 90% OR cant_drink = yes OR cant_sit = yes OR cyanosis = yes**			
stridor	Stridor (observed)	Does the child have stridor?	0, No<br>1, Yes
indrawing	Chest in-drawing (observed)	Does the child have chest indrawing?	0, No<br>1, Yes
flaring	Nasal flaring (observed)	Does the child have nasal flaring?	0, No<br>1, Yes
grunting	Grunting (observed)	Does the child have a grunt?	0, No<br>1, Yes
tracheal_tug	Tracheal tug (observed)	Does the child have a tracheal tug?	0, No<br>1, Yes
accessory_use	Accessory muscle use (observed)	Does the child use accessory muscles when breathing?	0, No<br>1, Yes
wheezing	Wheezing (observed)	Does the child have wheezing?	0, No<br>1, Yes
cough	Cough (observed)	Have you observed coughing from the child?	0, No<br>1, Yes
**Malnutrition**			
oedema	Visible oedema (feet, knees, face) (observed)	Does the child have pitting oedema on their feet, knees, or face?	0, No<br>1, Yes
**IF: muac < 125 mm OR age < 12 months**			
sev_wasting	Visible severe wasting (observed)	Does the child seem wasted?	0, No<br>1, Yes
**Infection**			
fever	History of fever (reported)	Ask: Does the child have a history of fever?	0, No<br>1, Yes
**IF age < 1 month**			
neo_jaundice	Neonatal jaundice (observed)	Does the child have yellowed sclera, palms, or soles?	0, No<br>1, Yes
umbilicus_red	Umbilicus - red (observed)	Is the child's umbilicus or surrounding skin red?	0, No<br>1, Yes
**IF umbilicus_red = yes**			
umbilicus_drain	Umbilicus- draining/pus (observed)	Does the child's umbilicus or surrounding skin have pus draining from it or trapped in it?	0, No<br>1, Yes
rash	Rash (observed)	Does the child have a rash?	0, No<br>1, Yes
**IF rash = yes**			
location_rash	Rash body part (observed)	Where is the rash located?	1, Generalized<br>2, Face<br>3, Neck<br>4, Torso<br>5, Arm<br>6, Hand<br>7, Leg<br>8, Foot<br>9, Genital<br>10, Perianal

lesion	Infective lesion (observed)	Does the child have an infective lesion on the skin, eye or ear?	0, No<br>1, Yes
**IF age < 1 month**			
oral_thrush	Oral thrush (observed)	Does the child have white patches on the roof of their mouth?	0, No<br>1, Yes
**IF temp ≥ 37.5°C OR fever = yes**			
neck_pain	Neck pain/stiffness (observed)	Does the child have pain on flexion of the neck?	0, No<br>1, Yes
bul_fontanelles	Bulging fontanelles (observed)	Does the child's fontanelles seem to bulge out above surrounding skin when lying supine and not crying?	0, No<br>1, Yes
muscle_tone	Abnormal muscle tone (observed)	Is the child's muscle tone abnormal? (see SOP)	0, No<br>1, Yes
**IF muscle_tone = yes**			
abnormal_tone	Type of abnormal muscle tone	Is the child's muscle tone increased or decreased? (see SOP)	1, Increased<br>2, Decreased
**Laboratory Testing**			
hiv	HIV testing	Enter HIV test result.	
malaria	Malaria test	Enter malaria test result.	
hb	Hemoglobin (g/dL)	Enter hemoglobin test result.	
hb_done	Hemoglobin test done?	Did the child receive a hemoglobin test today?	0, No<br>1, Yes
glucose	Blood sugar (mmol/L)	Enter blood glucose test result.	
glucose_done	Blood sugar test done?	Did the child receive a glucose test today?	0, No<br>1, Yes
lactate	Lactate (mmol/L)	Enter lactate test result.	
lactate_done	Lactate test done?	Did the child receive a lactate test today?	0, No<br>1, Yes
**IF neo_jaundice = yes**			
bilirubin	Bilirubin measurement (micromol/L)	Enter bilirubin test result.	
bilirubin_done	Bilirubin test done?	Did the child receive a bilirubin test today?	0, No<br>1, Yes
**Patient History**			
**Demographic Information**			
**KENYA RESIDENTIAL INFORMATION**			
district	District	Select district of residence from dropdown menu.	
county	County	Select county of residence from dropdown menu.	
division	Division	Select division of residence from dropdown menu.	
location	Location	Select location of residence from dropdown menu.	
sub_location	Sub location	Select sub location of residence from dropdown menu.	
village	Village or Estate	Select village/estate of residence from dropdown menu.	
**UGANDA RESIDENTIAL INFORMATION**			
district	District	Select district of residence from dropdown menu.	
county	County	Select county of residence from dropdown menu.	

sub_county	Sub-County	Select sub-county of residence from dropdown menu.	
parish	Parish	Select parish of residence from dropdown menu.	
village	Village	Select village of residence from dropdown menu.	
chairman	Chairman	Select chairman of residence from dropdown menu.	
**BOTH SITES**			
telephone_1	Telephone1	Enter a contact phone number.	
contact_1	Relationship to child	Ask: What is the relationship of this contact to the child?	
telephone_2	Telephone2	Enter a second contact phone number.	
contact_2	Relationship to child	Ask: What is the relationship of this contact to the child?	
telephone_3	Telephone2	Enter a third contact phone number.	
contact_3	Relationship to child	Ask: What is the relationship of this contact to the child?	
lo_illness	Length of Acute Illness (days, reported)	Ask: How many days has your child been sick in this illness?	
prev_admissions	Admission within 6 months (reported)	Ask: Has the child been admitted to a hospital or health center in the last 6 months?	0, No<br>1, Yes
**IF prev_admissions = yes**			
time_since_last_hosp	Time since last hospitalization (days, reported)	Ask: How long ago was the child last admitted to a hospital or health center?	1, <7 days<br>2, 7-14 days<br>3, 14-30 days<br>4, 1 month<br>5, 2 months<br>6, 3 months<br>7, 4 months<br>8, 5 months<br>9, 6 months
prev_hosps	Number of times hospitalized within 6 months (reported)	Ask: How many times was the child admitted to a hospital or health center in the last 6 months?	
urgent_referral	Referred within last 24 hours?	Ask: Was the child referred to this hospital from another health facility in the last 24 hours?	0, No<br>1, Yes
**Sociodemographic Information**			
primary_caregiver	Primary caregiver	Ask: Who is the primary caregiver of the child?	0, Mother<br>1, Father<br>2, Grandparent<br>3, Other relative<br>4, Non-relative<br>5, Day-care
**IF primary_caregiver NOT = mother**			
mother_alive	Mother alive	If mother not present, ask: Is the child's mother still alive?	0, No<br>1, Yes<br>98, Don't know
**IF (primary_caregiver NOT = mother AND mother_alive = yes) OR primary_caregiver = mother**			
maternal_age	Maternal age (years), if alive	Ask: How old is the child's mother?	
**IF age < 12 months**			
breastfeeding	Breastfeeding regimen (reported)	Ask: Was the child exclusively, partially or never breastfed for the first six months of life?	1, Exclusive<br>2, Partial<br>3, Never<br>98, Don't know

maternal_hiv	Maternal HIV status (reported)	Record HIV status if records available. Otherwise ask: Is the child's mother's HIV status?	0, Negative<br>1, Positive<br>98, Don't know
**IF maternal_hiv = positive**			
maternal_hiv_tx	Maternal HIV treatment (reported)	Ask: What HIV medication is the mother taking?	0, None<br>1, PMTCT<br>2, ART<br>98, Don't know
child_hiv	Child HIV status (reported)	Record HIV status if records available. Otherwise ask: Has your child tested positive for HIV?	0, No<br>1, Yes<br>98, Don't know
maternal_edu	Maternal education (reported)	Ask: What is the highest level of school completed by the child's mother? Read out options.	0, No school<br>1, Primary<br>2, Secondary<br>3, Post-secondary<br>98, Don't know
water_pure	All drinking water purified (reported)	Ask: Do you boil, filter (good sand/ceramic) or disinfect (using bleach/waterguard) all drinking water?	0, No<br>1, Yes
water_source	Primary water source for drinking (reported)	Ask: Where does your child get most of his/her drinking water from? Read out options.	1, Bottled<br>2, Tap/Municipal water<br>3, Bore hole<br>4, Protected spring<br>5, Open source (unprotected, stagnant water dam)<br>6, Slow running water<br>7, Fast running water<br>98, Don't know<br>99, Other
**IF water_source = other**			
water_source_other	Other primary water source for drinking	Ask: Where does your child get most of his/her drinking water from?	
**Pregnancy and Birth Information (children <12 months)**			
**IF age < 12 months**			
bw_known	Exact birth weight known	Check yes if record of birth weight is available. Otherwise ask: Do you know the child's weight when they were born?	0, No<br>1, Yes
**IF bw_known = yes**			
birth_weight	Exact birth weight (records, use reported if unavailable)	Enter birth weight from records if available. Otherwise ask: What was the child's weight when they were born?	
**IF bw_known = no**			
low_bw_est	Estimated low birth weight (reported)	Ask: Was the child diagnosed to have low birth weight when they were born?	0, No<br>1, Yes<br>98, Don't know
**IF age < 12 months**			
gestage_known	Exact gestational age known	Check yes if record of gestational age is available. Otherwise ask: Do you know how many weeks the pregnancy was when the child was born?	0, No<br>1, Yes
**IF gestage_known = yes**			
gestage	Gestational age at delivery (records, use reported if unavailable)	Enter gestational age at delivery in weeks from records if available.	

		Otherwise ask: How many weeks was the child when they were born?	
**IF gestage_known = no**			
gestage_est	Approximate gestational age in months (reported)	Ask: How many months (weeks) was the mom pregnant when the child was born?	0, < 7 (28)<br>1, 7-8 (28-32)<br>2, > 8 (32)<br>98, Don't know
**IF age < 12 months**			
facility_birth	Facility of birth (reported)	Ask: Where was the child born? Read out options.	1, Hospital<br>2, Health centre<br>3, Clinic<br>4, Home<br>5, Other<br>98, Don't know
**Other**			
parent_concern	Parental Concern	Ask: Do you think your child needs to be admitted to the hospital?	0, No<br>1, Yes<br>2, Healthcare workers should decide
**Outcomes**			
**Hospital Outcomes**			
*admitted	Hospital Admission	Was the participant admitted to the hospital?	0, No<br>1, Yes
**IF admitted = yes**			
diagnosis	Final Diagnosis	Look at the child's record to find the clinician diagnosis. Select the best category.	1, Malaria<br>2, Pneumonia<br>3, Bronchiolitis<br>4, URTI (cold, flu, etc)<br>5, Reactive airway disease/asthma<br>6, Gastroenteritis/Diarrhoea<br>7, HIV/AIDS or AIDS related illness<br>8, Meningitis/encephalitis or other CNS infection<br>9, Malnutrition<br>10, Tuberculosis<br>11, Any skin or soft tissue infection<br>12, Measles<br>99, Other
**IF diagnosis = other**			
diagnosis_other	Final Diagnosis Other	If diagnosis is 'other', please specify.	
**IF admitted = yes**			
*admitted_date	Date of Admission	Enter date of admission from record.	
*discharge_date	Date of Discharge	Enter date of discharge from record.	
*los	Length of Stay at Hospital	Calculated length of hospitalization.	
**Follow-up Call**			
*mortality	7-day Mortality Status	Find out whether the child is alive (See SOP).	0, Alive<br>1, Dead
**IF mortality = yes**			
*mort_inhosp	Died in hospital?	Ask: Did the child die in hospital?	0, No<br>1, Yes
confirm_admitted	Confirm Admission	Ask: Was your child admitted to Jinja hospital when we first met you?	0, No<br>1, Yes
**IF admitted = yes**			
confirm_admission	Confirm Date of Admission	Ask: What date was your child admitted to the hospital? (See SOP).	

confirm_discharge	Confirm Date of Discharge	Ask: What date was your child discharged from the hospital? (See SOP).	
*readmitted	7-day Readmission/admission to another hospital after discharge	Ask: Was the child admitted to any hospital after being discharged? (See SOP).	0, No<br>1, Yes
**IF readmitted = yes**			
readmitted_reason	Reason for readmission/admission to hospital after discharge	Ask: What was the reason the child was admitted?	1, Malaria<br>2, Pneumonia<br>3, Bronchiolitis<br>4, URTI (cold, flu, etc)<br>5, Reactive airway disease/asthma<br>6, Gastroenteritis/Diarrhoea<br>7, HIV/AIDS or AIDS related illness<br>8, Meningitis/encephalitis or other CNS infection<br>9, Malnutrition<br>10, Tuberculosis<br>11, Any skin or soft tissue infection<br>12, Measles<br>99, Other
*readmitted_location	Location of readmission/admission to hospital after discharge	Ask: Which hospital was the child admitted to?	
**IF readmitted_reason = other**			
reason_other	Readmission Reason Other	If reason for readmission is 'other', please specify.	
readmitted_tx	Treatment Received During Readmission	What treatment was the child given when readmitted?	0, None<br>1, Antibiotics<br>2, Intravenous Fluids<br>3, Oxygen<br>99, Other
**IF readmitted_tx = other**			
readmitted_tx_other	Treatment Received During Readmission Other	If treatment is 'other', please specify.	
*readmitted_date_known	Readmission dates known	Do you know exactly what dates the child was admitted again and discharged again from the hospital?	0, No<br>1, Yes
**IF readmitted_date_known = yes**			
*readmitted_start	Readmission date	What date was the child admitted again from the hospital after being sent home?	
*readmitted_end	Readmission discharge date	What date was the child sent home again after being readmitted?	
*readmitted_los_calc	Length of stay on readmission (calculated)	Calculated length of stay on readmission	
**IF readmitted_date_known = no**			
*readmitted_los_reported	Length of stay on readmission (reported)	When your child was admitted to the hospital again, how many days did your child stay in hospital?	
**IF readmitted = yes**			
*seek_help_before_readmit	Seeking help at medical facility before readmission	Ask: Other than the place where your child was readmitted, did you seek any other medical help?	0, No<br>1, Yes

**IF readmitted = no**			
*seek_help	Seeking help at medical facility (regardless of readmission)	Ask: Did you seek help from a medical facility after being discharged from hospital?	0, No<br>1, Yes
**Clinical Timekeeper**			
study_id	Study ID	Enter the study ID recorded in the participant's notebook.	
study_id_2	Study ID	Again, enter the study ID recorded in the participant's notebook.	
*abx_time	Time at start of antibiotics administration.	Time at start of antibiotics administration.	
*fluids_time	Time at start of intravenous fluids administration.	Time at start of intravenous fluids administration.	
*oxygen_time	Time at start of oxygen administration.	Time at start of oxygen administration.	
other_time	Time at start of treatments that are NOT antibiotics, fluids or oxygen.	Time at start of treatments that are NOT antibiotics, fluids or oxygen.	
**IF tx = other**			
tx_other	Treatment Received Other	If treatment is 'other', please specify.	
