# Supplementary material for: Smart triage: triage and management of sepsis in children using the point-of-care Pediatric Rapid Sepsis Trigger (PRST) tool
Source: BMC Health Serv Res. 2020 Jun 3;20:493. doi: 10.1186/s12913-020-05344-w (PMC7268489; doi:10.1186/s12913-020-05344-w)
Supplement: Supplementary file 2 — Additional file 2. Triage Tool Training Questionnaire. [file 12913_2020_5344_MOESM2_ESM.pdf]

Smart Triage: Triage and management of sepsis in children using the point-of care Paediatric Rapid Sepsis Trigger (PRST) tool

TRIAGE TOOL TRAINING QUESTIONNAIRE

Version 1.0

Participant ID: ______________________________ Date: _____________ Time: __________

Impact

Question #1: Do you think the triage tool would be a positive addition to the patient risk assessment process at triage?

Response:

____
____
____
____

Question #2: Do you think the triage tool would improve the patient risk assessment processes at your hospital?

Response:

____
____
____
____

Question #3: Do you think the triage tool would be an important part of meeting your information needs related to patient risk levels?

Response:

____
____
____
____

Perceived Usefulness

Question #4: Do you think using the triage tool makes it easier for you to identify patients who may be at higher risk and need more attention?

Response:

____

____

Question #5: Do you think the triage tool enables you to identify higher risk patients more quickly?

Response:

Question #6: Do you think using the triage tool makes it more likely that you will identify patients who are higher risk at triage?

Response:

Question #7: Do you think that using the triage tool is useful for identifying high risk patients at triage?

Response:

Question #8: Do you think the triage tool presents a more equitable process for patient risk assessment at triage?

Response:

____
____
____

Question #9: Are you satisfied with the triage tool for risk management of patients at triage?

Response:

____
____
____
____

Question #10: Do you think you will be able to manage patients risk assessment at triage in a timely manner because of the triage tool?

Response:

____
____
____
____

Question #11: Do you think using the triage tool increases your ability to assess patients' risk levels at triage?

Response:

____
____
____
____

Question #12: Are you able to assess patient risk at triage whenever you use the triage tool?

Response:

____
____

____

____

Perceived Ease of Use

Question #13: Are you comfortable with your ability to use the new triage tool?

Response:

____

____

____

____

Question #14: Was learning to operate the triage tool easy for you?

Response:

____

____

____

____

Question #15: Was it easy for you to become skillful at using the triage tool?

Response:

____

____

____

____

Question #16: Do you find the triage tool easy to use?

Response:

____

____

____

____

Question #17: Can you always remember how to log on to and use the triage tool?

Response:

____

____

____

____

User Control

Question #18: Do you find the triage tool gives error messages that clearly tell you how to fix problems?

Response:

____

____

____

____

Question #19: When you make a mistake using the triage tool, do you find you can recover easily and quickly?

Response:

____

____

____

____

Question #20: Do you think the information (such as on-screen messages and other documentation) provided with the triage tool is clear?

Response:

____

____

____

____

Healthcare Worker Satisfaction

Question #21: Do you feel like you are well trained and prepped to use the triage tool and standard protocols when they are implemented in the hospital?

Response:

____
____
____
____

Question #22: Overall, do you think the triage tool will be a positive addition to your hospital?

Response:

____
____
____
____

Additional Comments

Please enter any additional notes you have for us.

____
____
____
____

Your input and comments are an important part of evaluating the tools and training of this program. Thank you!
