# Supplementary material for: Smart triage: triage and management of sepsis in children using the point-of-care Pediatric Rapid Sepsis Trigger (PRST) tool
Source: BMC Health Serv Res. 2020 Jun 3;20:493. doi: 10.1186/s12913-020-05344-w (PMC7268489; doi:10.1186/s12913-020-05344-w)
Supplement: Supplementary file 3 — Additional file 3. Healthcare Worker Satisfaction Survey. [file 12913_2020_5344_MOESM3_ESM.pdf]

Smart Triage: Triage and management of sepsis in children using the point-of care Paediatric Rapid Sepsis Trigger (PRST) tool

HEALTHCARE WORKER SATISFACTION SURVEY

Version 1.0

Participant ID: ______________________ Date: __________ Time: ________

Healthcare Worker Satisfaction – Overall

Question #1: Do you feel you have adequate opportunities to develop your professional skills?

Response:

____
____
____
____

Question #2: Do you feel the amount of work you are expected to finish each week is reasonable?

Response:

____
____
____
____

Question #3: Do you feel your department provides all the equipment, supplies and resources necessary for you to perform your duties?

Response:

____
____
____
____

Question #4: What percent of the time do you feel confident in your skills at your work?

Response:

____
____
____
____

Question #5: Do you feel that you work well with your coworkers?

Response:

____
____
____
____

Question #6: Do you feel you can easily communicate with members from all levels of this organization?

Response:

____
____
____
____

Question #7: Would you recommend this health facility to other workers as a good place to work?

Response:

____
____
____
____

Impact

Question #8: Do you think the triage tool was a positive addition to the patient risk assessment process at triage?

Response:

____
____
____
____

Question #9: Do you think the triage tool improved the patient risk assessment processes at your hospital?

Response:

____
____
____
____

Question #10: Do you think the triage tool provides adequate information required to prioritize your patients?

Response:

____
____
____
____

Perceived Usefulness

Question #11: Did you find using the triage tool made it easier for you to identify patients who may be at higher risk and need additional attention?

Response:

____
____
____
____

Question #12: Did the triage tool enable you to identify higher risk patients more quickly?

Response:

____
____

____
____

Question #13: Did the triage tool make it more likely that you would identify patients who are at higher risk at triage?

Response:

____
____
____
____

Question #14: Do you think the triage tool and standard protocols allow for a more equitable (fair) process to assess risk assessment at triage?

Response:

____
____
____
____

Question #15: Are you satisfied with the triage tool for risk management of patients at triage?

Response:

____
____
____
____

Question #16: Do you find that you manage patients' assessments at triage in a timely manner because of the triage tool?

Response:

____
____
____
____

Question #17: Are you able to assess patient risk at triage whenever you use the triage tool?

Response:

____
____
____
____

Perceived Ease of Use

Question #18: Are you comfortable with your ability to use the new triage tool?

Response:

____
____
____
____

Question #19: Did you feel you were properly trained to use the triage tool?

Response:

____
____
____
____

Question #19: Was learning to use the triage tool easy for you?

Response:

____
____
____
____

Question #20: Was it easy for you to become skillful at using the triage tool?

Response:

____
____
____
____

Question #21: Do you find the triage tool easy to use?

Response:

____
____
____
____

Healthcare Worker Satisfaction

Question #22: Overall, do you feel the triage tool helped processes in the outpatient department?

Response:

____
____
____
____

Question #23: Overall, do you think the information provided on the triage tool is clear?

Response:

____
____
____
____

Question #24: Do you think the triage tool has improved wait times in the outpatient department?

Response:

____
____
____
____

Question #25: Do you feel that you were well trained and prepared before the triage tool was implemented in the hospital?

Response:

____
____
____
____

Question #26: Do you feel the triage tool helped healthcare workers in the OPD to better communicate with each other?

Response:

____
____
____
____

Question #27: Overall, do you think the triage tool was a positive addition to your hospital?

Response:

____
____
____
____

Additional Comments

Please enter any additional notes you have for us.

____
____

____

____

Your input and comments are an important part of evaluating the tools and training of this program. Thank you!
